# The effect of stimulus context on the buildup to stream segregation

**DOI:** 10.3389/fnins.2014.00093

**Published:** 2014-04-29

**Authors:** Jonathan Sussman-Fort, Elyse Sussman

**Affiliations:** ^1^Department of Neuroscience, Albert Einstein College of Medicine, Yeshiva UniversityBronx, NY, USA; ^2^Department of Otorhinolaryngology-Head and Neck Surgery, Albert Einstein College of Medicine, Yeshiva UniversityBronx, NY, USA

**Keywords:** auditory perception, mismatch negativity, stream segregation, event-related potentials, auditory scene analysis

## Abstract

Stream segregation is the process by which the auditory system disentangles the mixture of sound inputs into discrete sources that cohere across time. The length of time required for this to occur is termed the “buildup” period. In the current study, we used the buildup period as an index of how quickly sounds are segregated into constituent parts. Specifically, we tested the hypothesis that stimulus context impacts the timing of the buildup and, therefore, affects when stream segregation is detected. To measure the timing of the buildup we recorded the Mismatch Negativity component (MMN) of event-related brain potentials (ERPs), during passive listening, to determine when the streams were neurophysiologically segregated. In each condition, a pattern of repeating low (L) and high (H) tones (L-L-H) was presented in trains of stimuli separated by silence, with the H tones forming a simple intensity oddball paradigm and the L tones serving as distractors. To determine the timing of the buildup, probe tones occurred in two positions of the trains, early (within the buildup period) and late (past the buildup period). The context was manipulated by presenting roving vs. non-roving frequencies across trains in two conditions. MMNs were elicited by intensity probe tones in the Non-Roving condition (early and late positions) and the Roving condition (late position only) indicating that neurophysiologic segregation occurred faster in the Non-Roving condition. This suggests a shorter buildup period when frequency was repeated from train to train. Overall, our results demonstrate that the dynamics of the environment influence the way in which the auditory system extracts regularities from the input. The results support the hypothesis that the buildup to segregation is highly dependent upon stimulus context and that the auditory system works to maintain a consistent representation of the environment when no new information suggests that reanalyzing the scene is necessary.

## Introduction

When entering a noisy scene, such as a crowded sports arena, the auditory system is faced with the “problem” of parsing out the mixture of sounds to their distinct sources, a process that has been termed “auditory scene analysis” (Bregman, 1990). Bregman (1978) hypothesized that the mixture of sounds entering the ears is initially perceived as integrated. Only after some time in which information about the sound characteristics accumulates would the mixture of sounds be perceived as distinct sound streams. This was supported by Anstis and Saida (1985) who found that there was a higher probability that listeners' perceived a sound mixture as segregated as time elapsed over a 30 s trial. The time that it takes to perceive an integrated mixture of sounds as segregated streams has been called the “buildup” period. In the current study, we used the buildup period as an index of how quickly sounds are segregated into constituent parts.

Most previous studies have tested the buildup to stream segregation by manipulating the frequency distance between two sets of sounds and measuring the time it takes to indicate that two streams are perceived from the onset of the sound sequence. Using this approach, studies demonstrated that the buildup to perceiving segregated streams occurred faster the larger the frequency distance between sounds or the more rapid the stimulus presentation rate (Bregman, 1978; Cusack et al., 2004; Micheyl et al., 2005; Snyder et al., 2008; Haywood and Roberts, 2010). However, in more natural listening scenes, sound features are often dynamically changing. We hypothesize that the extent of change in the sound input contributes to how quickly we adapt to the auditory scene. That is, the buildup period should be longer when the scene is more dynamic, in that more information would be needed to make a decision about whether a sequence is segregated or not. In this study, we test the influence of changing parameters on stream segregation by measuring the timing of the buildup to detection of stream segregation when the input is stable vs. when it is changing. In this first step of addressing our hypothesis, we took the influence of attention out of the equation, and measured the timing to stream segregation using a neurophysiologic index that we have used previously (Sussman et al., 2007).

There has been some debate about the role of attention in the formation of auditory streams (Jones et al., 1999; Carlyon et al., 2001; Cusack et al., 2004; Sussman et al., 2007) but certainly there is evidence that attention modulates the perceptual decision about stream segregation (Jones et al., 1999; Carlyon et al., 2001; Cusack et al., 2004; Snyder et al., 2006). One issue with attention is whether the behavioral response is measuring the buildup, or the time to perceptual decision (Deike et al., 2012). Additionally, performing a task or attending to a subset of sounds may influence threshold levels for detecting segregation (Sussman et al., 1998a, 2005; Sussman, 2007; Sussman and Steinschneider, 2009). In the current study, we used an index of change detection to assess the timing of when sounds were neurophysiologically segregated without the influence of task performance. This allowed us to assess the influence of stimulus context on the timing of the buildup, if it exists, without attentional manipulation.

To test our hypothesis, we recorded event-related brain potentials (ERPs), and measured the mismatch negativity (MMN) component. The MMN is an appropriate tool because it can index sound change detection irrespective of the direction of attention (Sussman et al., 2003; Winkler et al., 2005). MMN is generated bilaterally within auditory cortices and the negative waveform is observed with a fronto-central scalp distribution that inverts in polarity at the mastoid electrodes (Vaughan and Ritter, 1970; Giard et al., 2014). MMN is elicited by detected infrequent deviant tones presented among a series of more frequently presented standard tones. The standard-to-deviant relationship can be set up by repetition of any tone feature (e.g., frequency, intensity, or duration). Thus, in the simplest case, the MMN component is the end result of a comparison process between the frequently presented standard tone and detection of a deviant feature. In the current study, we used intensity deviants to elicit MMN in an oddball stream that emerges only when the sounds are neurophysiologically segregated (Sussman et al., 2007; Sussman and Steinschneider, 2009). Thus, the ability to detect the intensity deviants (herein we will call them “probe tones” because they are not always detected as deviants), and the elicitation of the MMN in the current study, indicates neurophysiologic segregation of sounds (see Methods for details).

Sussman et al. (2007) found evidence that there was a buildup to stream segregation even when no task was performed with the sounds, when participants were watching a movie and reading closed-captions. In that study, trains of low (L) and high (H) tones were presented with probe tones randomly embedded during the supposed buildup period (in the beginning of the train of tones), as well as randomly placed after the buildup period was surpassed (in the later part of the train). The MMN was elicited by intensity deviants in an oddball stream by probe tones only in the later part of the train. The absence of MMN to probe tones embedded in the beginning of the trains indicated that the probes occurred before the L and H tones were neurophysiologically segregated (i.e., during the buildup period). No intensity regularity was detected during that time period. However, the study design could be considered as a factor in the results, in that the trains of tones roved in frequency across 10 different frequency levels. There was no repetition of tone frequency from train-to-train even though the semitone (ST) distance between trains was the same for all trains (8 ST). This roving frequency leaves open the question of whether the occurrence of each new train of an unpredicted frequency value may have signaled a new “event.” That is, it is possible that the roving frequency may have initiated the analysis of stream segregation for each train, and precluded any carryover effects that may have occurred if each train had the same tone frequencies as the previous ones. If, on the other hand, one could tell at the beginning of the train that it was the same as the one that occurred before it (i.e., the same event occurred again after the silence), we hypothesized for the current study that the predictability or repetition could then affect the timing of the buildup (i.e., faster to segregation when each train could be anticipated). Thus, we predicted that the timing of segregation, as indexed by elicitation of the MMN to probe tones randomly occurring in the beginning of the tone trains, would be significantly altered by the stimulus context (whether the train-to-train frequency was repeated). To test this, we replicated a condition from Sussman et al. (2007) that roved trains by frequency (Roving condition) and compared it to a condition in which the tone frequency of the trains was repeated (Non-Roving condition). If the buildup could be modulated by stimulus context, whether the tone trains were repeated or roved in frequency, we predicted that it would be faster when there was repetition of tone frequency than when there was not, and thus elicitation of MMN to probe tones occurring in the beginning part of the tone trains, during the buildup period, should differ between the two conditions.

## Materials and methods

### Subjects

Fifteen adults, 6 males (22–40 years) (*M* = 30 years, *SD* = 4.8 years) were paid for their participation in the study. All participants passed a hearing screen (20 dB HL at 500, 1000, 2000, and 4000 Hz), and had no reported history of neurological disorders. One participant's data were excluded due to excessive eye artifacts. The data from the remaining 14 participants were included in the analysis and are reported. All procedures were approved by the Albert Einstein College of Medicine Internal Review Board and were conducted in accordance with the Declaration of Helsinki. Informed consent was obtained from all participants after the procedures were explained to them.

### Stimuli and procedures

Complex tones (five harmonics above the fundamental frequency) were created using Adobe Audition 1.0 (Adobe Systems Inc., San Jose, CA). Tone duration was 30 ms (including a 5 ms rise/fall time). Table 1 displays the five different tone pairs used in the study. Each column represents one tone pair and is labeled “A”–“E.” Hereforth, individual tones will be referred to by their fundamental frequency shown in Table 1. Tones were presented bilaterally via insert earphones (E-A-RTone 3A; Indianapolis, IN) using Neuroscan STIM hardware and software (Compumedics Inc., Charlotte, NC). Sounds were calibrated using a Bruel and Kjaer 2209 (Denmark) impulse precision sound pressure level meter with an artificial ear by presenting each complex tone to the meter and adjusting for any difference in the actual sound level measured.

**Table 1:** **Fundamental frequencies of the tones**.

**Tone pair**	**A**	**B**	**C**	**D**	**E**
Low (Hz)	329.83	440.00	587.33	783.99	1046.50
High (Hz)	523.35	698.25	932.33	1244.50	1661.20

**Figure 1:**
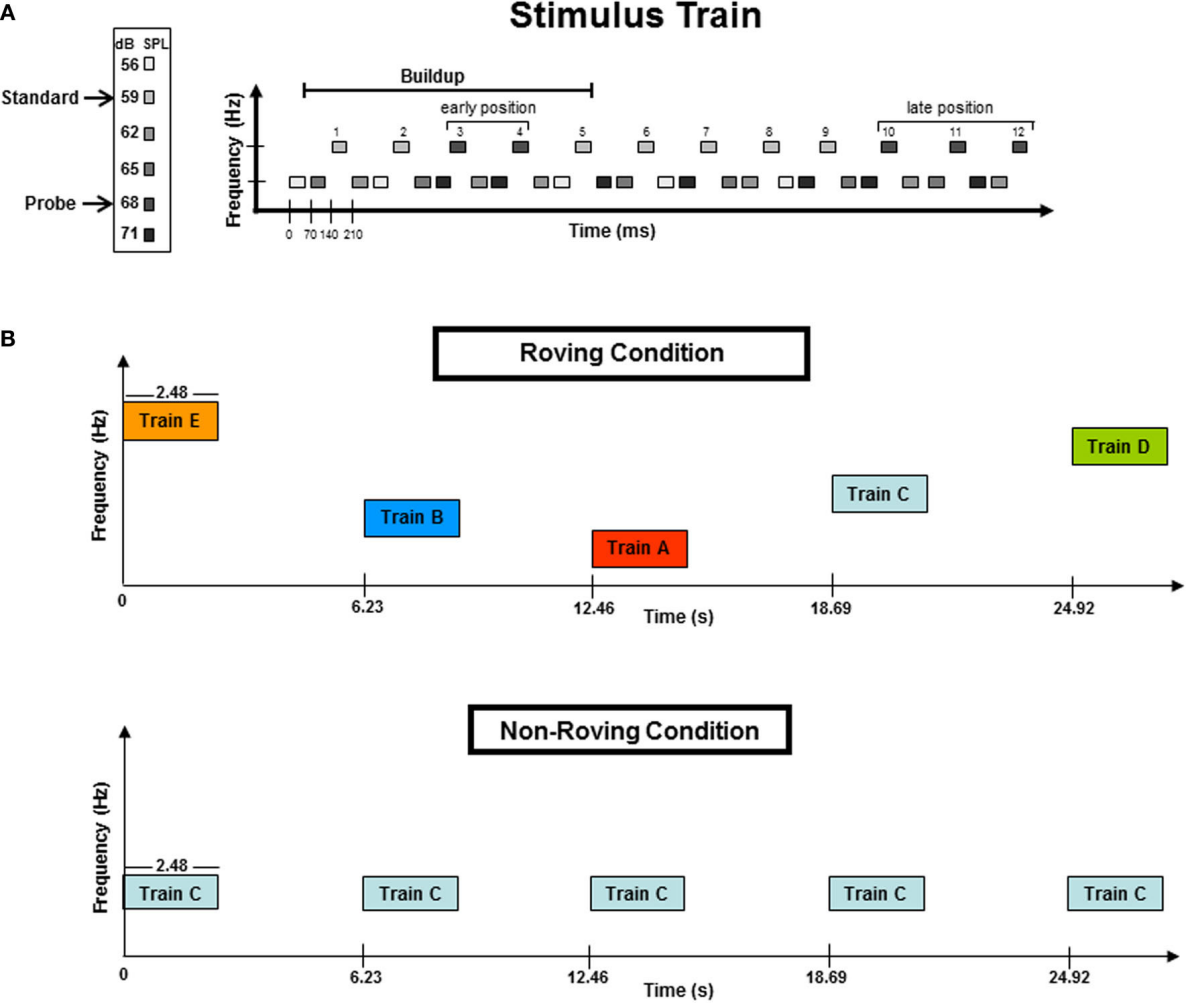
**(A)** A stimulus train consisted of 36 tones: 24 lower (L) frequency tones and 12 higher (H) frequency tones presented in a three-tone repeating sequence of L-L-H. The frequency separation between the H and L tones was eight semitones (ST). The H tones formed a simple intensity oddball with early position probe tones randomized across 3rd or 4th tones in the train. The buildup period, designated by previous studies, is delineated with a horizontal line. Late position probe tones were randomized in the 10th, 11th, or 12th tones of the trains. The late position tones occurred outside (after) the buildup interval. Frequency (Hz) is represented on the vertical axis while the horizontal axis represents time (ms). **(B)** Schematic of the Roving and Non-Roving conditions. The Roving condition (top panel) depicts the five frequency levels (A–E, see Methods for further details) randomly distributed across trains within a stimulus block. The ST distance was 8-ST for all five frequency levels. In the Non-Roving condition (bottom panel), only one frequency level was presented throughout each stimulus block (here represented by Train C). Frequency levels were randomized across stimulus blocks (not trains). All five frequency levels (A–E) were used in both conditions. The vertical axis represents frequency (Hz) and the horizontal axis represents time (in seconds). Train duration was 2.48 s. Intertrain interval (ITI) was 3.75 s of silence.

Figure 1 displays the stimulus paradigm. Tones were presented in blocks of 50 trains. Each train consisted of 36 tones, with 24 lower frequency tones (L) and 12 higher frequency tones (H) presented in a repeated three-tone sequence of L-L-H (Figure 1A). Stimulus onset asynchrony (SOA) was 70 ms, with an intertrain interval (ITI) of 3.75 s (Figure 1B). The Δ*f* of each frequency pair was eight semitones (ST). This frequency distance was chosen because it has previously been shown to induce streaming in passive listening conditions (Sussman et al., 2007; Sussman and Steinschneider, 2009). Tone intensity was used to elicit the MMN component within the high stream oddball sequence (Figure 1A), which occurs only when the tones neurophysiologically segregate (Sussman et al., 2001). To do this, tone intensity of the H tones was 59 dB SPL, with randomly and infrequently (17%) occurring “probe” tones that had a louder intensity value (68 dB SPL) (Figure 1A). The L tones were randomly assigned with four different stimulus intensities (56, 62, 65, and 71 dB SPL) that spanned above and below the intensity values of the H tones. This was done so that the oddball standard and deviant intensity tones had neither the loudest or softest intensity values within the overall tone train. Therefore, only when the tones neurophysiologically segregated would the within-stream oddball intensity relationship be detected, and have the possibility to elicit MMN. In contrast, if the tones did not neurophysiologically segregate, there was no standard intensity value to be detected, from which any tone could be detected as deviant. Therefore, the presence of the MMN component indicated segregation.

To test the hypothesis that the dynamics of the tone context can alter the timing of the buildup to segregation, probe tones were placed in both “early” and “late” positions within the trains (Figure 1A). Probe tones occurred randomly in either the third or the fourth tone position within the H tone stream (these were “early” probe tones), and randomly in the 10th, 11th, or 12th tone position within the H stream (these were “late” probe tones). Probe tone position was randomized across trains so that the position within the train could not be anticipated for either the early or late positions. The early position probe tones occurred within the buildup period, whereas the late position tones occurred after the buildup period was surpassed based on results of previous psychophysical and ERP studies (Cusack et al., 2004; Sussman et al., 2007). Thus, responses to the early position probe tones would indicate the timing of the buildup, as they were expected to elicit MMN only when the H and L tones were neurophysiologically segregated in memory at the time the probe tones occurred. All late position probe tones were expected to elicit MMN, and these served as a control, so that if no MMN were elicited in the beginning of the trains, MMN elicited by probe tones at the end of the train would provide evidence that the buildup had been surpassed.

### Procedures

Participants were seated in a comfortable chair in a sound-attenuated booth [Industrial Acoustics Company (IAC), Bronx, NY]. Two conditions were presented: Roving and Non-Roving (Figure 1B). In the Roving condition, the frequency values of the tones roved on five levels, randomly across trains within the stimulus block (Figure 1B, top panel). This was done so that there was no expectation of frequency value, no carryover from one train to the next. In the Non-Roving condition, all five tone pairs were used (i.e., A–E, as in the Roving condition) except that each block contained only one tone pair throughout. Thus, in the Non-roving condition, the tone frequency that occurred from train to train was fully expected. The 3.75 s ITI was chosen because it has been shown to reset the segregation process following that length of silence (Bregman, 1978; Cusack et al., 2004; Sussman et al., 2007); thus, each train served as a new trial for observing the timing of the buildup. Half of participants were presented with the Roving condition first, and the other half with the Non-roving condition first. The order of stimulus blocks within each condition was randomized across participants using a Latin Squares design. Additionally, control blocks were conducted to obtain comparison stimuli for the probe tones. In the control blocks, the intensity value of the standard and the probe tones in the high tone stream were reversed so that the standard tone intensity level was 68 dB and the probe tone was the softer 59 dB, without any other changes in the stimulus parameters. Thus, to delineate the MMN, we subtracted the ERP elicited by the standard tone obtained in the control block from the ERP elicited by the probe tone in the experimental blocks, contrasting two tones that had the same physical properties but differed only in the status within the train. (i.e., 68 dB probe-minus-68 dB standard) (Sussman et al., 2007). Twelve total blocks were presented, which includes the two control blocks, and each block was 5 min in length. Participants were instructed to watch a closed-captioned video and had no task with the sounds. The duration of the session was approximately 2.5 h, which included electrode cap placement and breaks.

### Data acquisition

Electroencephalogram (EEG) was recorded using a 32-electrode cap including a subset of the International 10–20 System (Jasper, 1958) (FPz, Fz, Cz, Pz, Oz, FP1, FP2, F3, F4, F7, F8, FC5, FC6, FC1, FC2, T7, T8, C3, C4, CP5, CP6, CP1, CP2, P7, P8, P3, P4, O1, O2) and left and right mastoids (LM and RM, respectively). The reference electrode was positioned on the tip of the nose. Data was digitized at a 500 Hz sampling rate (bandpass 0.05–100 Hz) (Neuroscan Synamps, Compumedics Inc., Charlotte, NC). Eye blinks were recorded using a bipolar configuration (VEOG) with an external electrode positioned below the left eye (EOG) and the FP1 electrode. Eye saccades were monitored using a bipolar configuration (HEOG) between the F7 and F8 electrodes. Impedances for each electrode were kept below 5 kOhms. Throughout the experiment the EEG was monitored for excessive movement and for eye saccades to ensure the participant was reading the captions.

### Data analysis

Post-processing of the data included bandpass filtering on the continuous recordings of the EEG offline from 1 to 15 Hz using a zero phase shift Butterworth filter and 24 dB/octave rolloff. Epochs were then created, 600 ms long, including a 100 ms pre-stimulus onset period. Each epoch was baseline corrected across this entire epoch before artifact rejection (±75 μV), then baseline corrected again using the prestimulus period.

ERP responses to the early 3rd and 4th position probe tones were averaged separately for both Roving and Non-Roving conditions. These were the main dependent measures used to evaluate the timing of the buildup to stream segregation. The ERP responses to the 10th, 11th, and 12th position probe tones were averaged together, and served as the “late position” deviant response separately in each condition. The 68 dB high tones were averaged together from the control blocks and served as the standard comparison, separately in each condition. Separately in both Roving and Non-Roving conditions, the averaged ERP responses to the control and to the probe tones were collapsed across all five frequency levels (train types A–E).

The MMN component was delineated in the grand averaged difference waveforms (average ERP elicited by the probe tone minus average ERP elicited by the control). To statistically evaluate the presence of the MMN, a 30-ms interval centered on the peak of the MMN was used to obtain the mean amplitudes evoked by the control and probe tones. The peak was determined in the difference waveforms in the Non-Roving condition from the 4th position and late position, where MMN peaks were observed. These peaks defined the intervals used to measure the MMN in the early and late positions in both conditions (Table 2). A two-way repeated measures analysis of variance (ANOVA) with factors of position (early 3rd, early 4th, late) and stimulus type (probe, control) was calculated (Statistica 10, Statsoft Inc., Tulsa, OK) to statistically compare the mean amplitudes in each condition. Huynh-Feldt corrections were applied for violations of sphericity and corrected *p*-values reported. *Post-hoc* tests were calculated using the Tukey HSD. Voltage maps of the scalp distribution were created from the peak latency used to measure the MMN in each condition and position, using BESA Research 6.0 software (BESA GmbH, Gräfelfing, Germany).

**Table 2:** **Mean amplitudes and intervals used to measure the MMN in each condition and position at the Fz electrode**.

**Condition**	**Position**	**Interval (ms)**	**Mean (std) μV**
Roving	3rd	115–145	0.66 (0.04)
	4th		−0.29 (0.16)
	Late	130–160	−0.58 (0.02)^*^
Non-Roving	3rd	115–145	0.06 (0.04)
	4th		−1.09 (0.10)^*^
	Late	130–160	−1.03 (0.08)^*^

* Indicates significant MMN.

To compare amplitude of the significant MMNs (Roving late, Non-Roving early 4th, and Non-Roving late position), the mean amplitudes of the difference waveforms were used in a one-way repeated measures ANOVA (Table 2).

To compare peak latencies of the significant MMNs (early 4th vs. late) in the Non-Roving condition, latencies were first determined by identifying the local minima [within a 90 ms window (85–175 ms for early probe tones, and 100–190 ms for late probe tones)] on each difference waveform, in each individual. Latency was then compared using a Students *t*-test for dependent measures.

## Results

**Figure 2:**
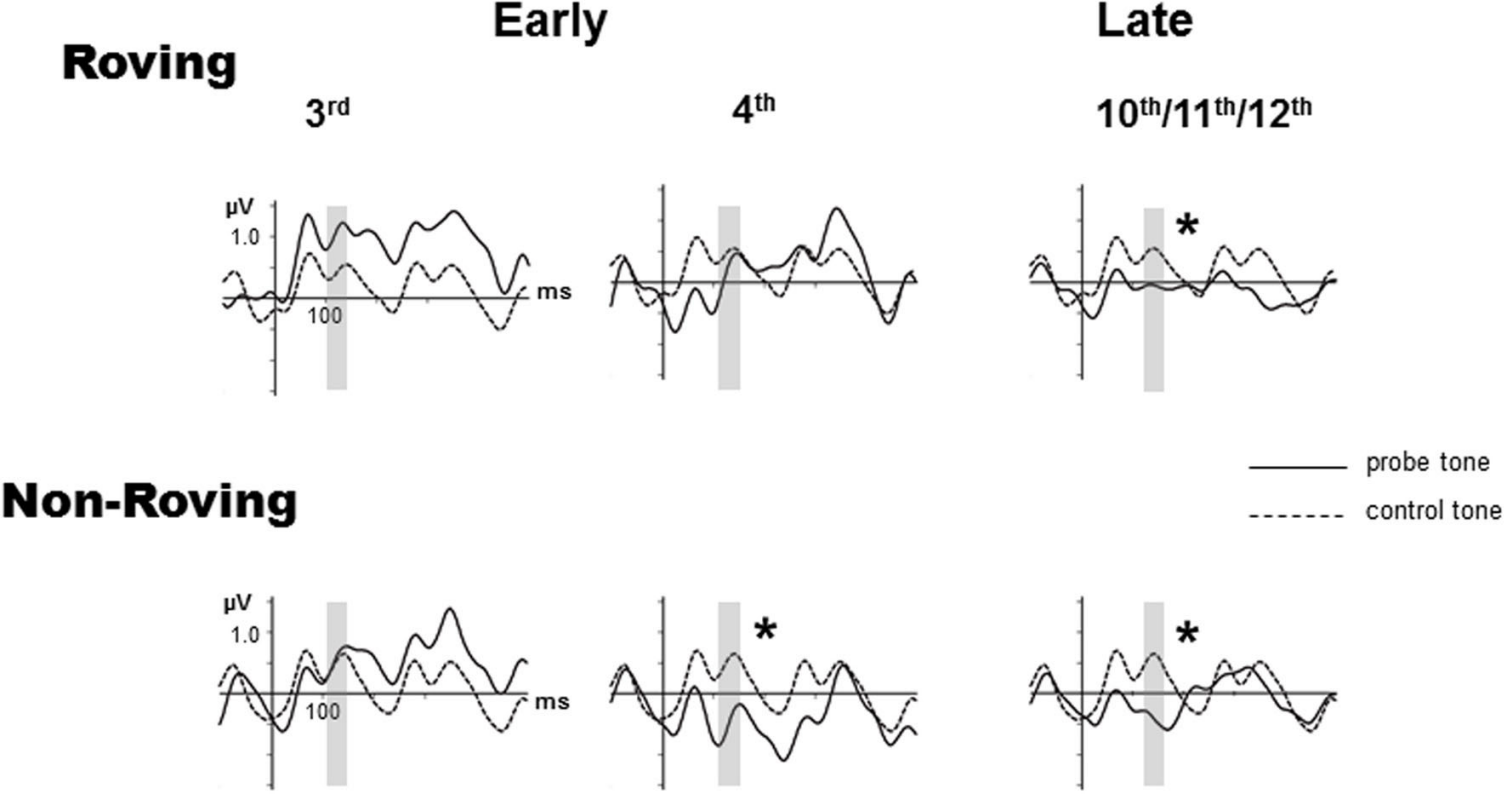
**Grand-averaged event-related potentials (ERPs) evoked by probe tones (solid line) and control tones (dashed line) are overlain at the Fz electrode for the early 3rd position tone (left column), early 4th position tone (middle column), and the late position tone, which is the average of the 10–12th tones (right column), separately for the Roving (top row) and Non-Roving (bottom row) conditions**. The vertical axis is positioned at stimulus onset, with a 100 ms prestimulus baseline and 500 ms post-stimulus epoch. The interval used to statistically measure the MMN is shaded in gray. Significant MMNs are marked with an asterisk.

Figure 2 displays the grand-averaged ERPs elicited by the probe and control stimuli. The P1 and the N1 components are small (peak latencies at 50 and 100 ms, respectively) due to the rapid 70 ms SOA presentation rate used in this study (Näätänen and Picton, 1987; Sussman et al., 1998b), but are nonetheless distinctly visible in the standard and deviant ERPs. The 600 ms epoch displays the responses to multiple successive stimuli that are visible and overlap in the waveform after tone onset. The Roving condition, due to the stochastic nature of the frequencies across trains within stimulus blocks shows the greatest variability throughout the epoch. This can be seen as larger differences in the responses to probe vs. control tones early in the epoch before the expected latency (100–250 ms) for MMN (Näätänen, 1995). For this reason MMN was not considered to be present prior to 100 ms.

In the *Roving condition*, there was no main effect of stimulus type [*F*_(1, 13)_ < 1, *p* = 0.68]. There was a main effect of position [*F*_(2, 26)_ = 3.65, ε = 0.87, *p* = 0.047]. *Post-hoc* analysis showed that the response to the late position probe tone was more negative than the responses to the early 3rd and early 4th position probe tones, with no difference between the 3rd and the 4th position. There was a significant interaction between position and stimulus type [*F*_(2, 26)_ = 3.92, ε = 0.90, *p* = 0.038]. *Post-hoc* analysis revealed that the response to the probe tone was more negative than the response to the control tone only for the late position stimuli, but not between the control and probe tone responses in either of the early position stimuli. There was no difference in response to the control tones by position. Thus, the difference was solely due to the response to the late position probe tone. These results show that MMN was significantly elicited (significant difference between probe and control tone) by the late position, but not either early position probe tones.

In the *Non-Roving condition*, there was a main effect of stimulus type [*F*_(1, 13)_ = 11.28, *p* = 0.005]. Overall, the ERP response to the probe tone was more negative than the ERP response to the control. There was also a main effect of position [*F*_(2, 26)_ = 9.34, ε = 0.87, *p* = 0.002]. *Post-hoc* analysis showed that the responses to the early 4th and late position probe tones were both more negative than the response to the early 3rd position probe tone, with no significant difference between the early 4th and late position probe tones. There was a significant interaction between position and stimulus type [*F*_(2, 26)_ = 9.59, ε = 0.94, *p* = 0.001]. *Post-hoc* analysis showed that the probe tone was significantly more negative than the control tone for the early 4th and late position, but not for the early 3rd position. There were no significant differences in the response to the control tones. These results show that MMN was significantly elicited by the early 4th and late position probe tones, but not by the early 3rd position probe tone.

Table 2 displays the mean amplitudes of the MMNs. The amplitudes of the significant MMNs did not significantly differ from each other [*F*_(2, 26)_ = 1.66, *p* = 0.21]. Peak latencies of the significant MMNs in the Non-Roving condition also did not significantly differ from each other [*t*_(13)_ = 0.62, *p* = 0.55].

**Figure 3:**
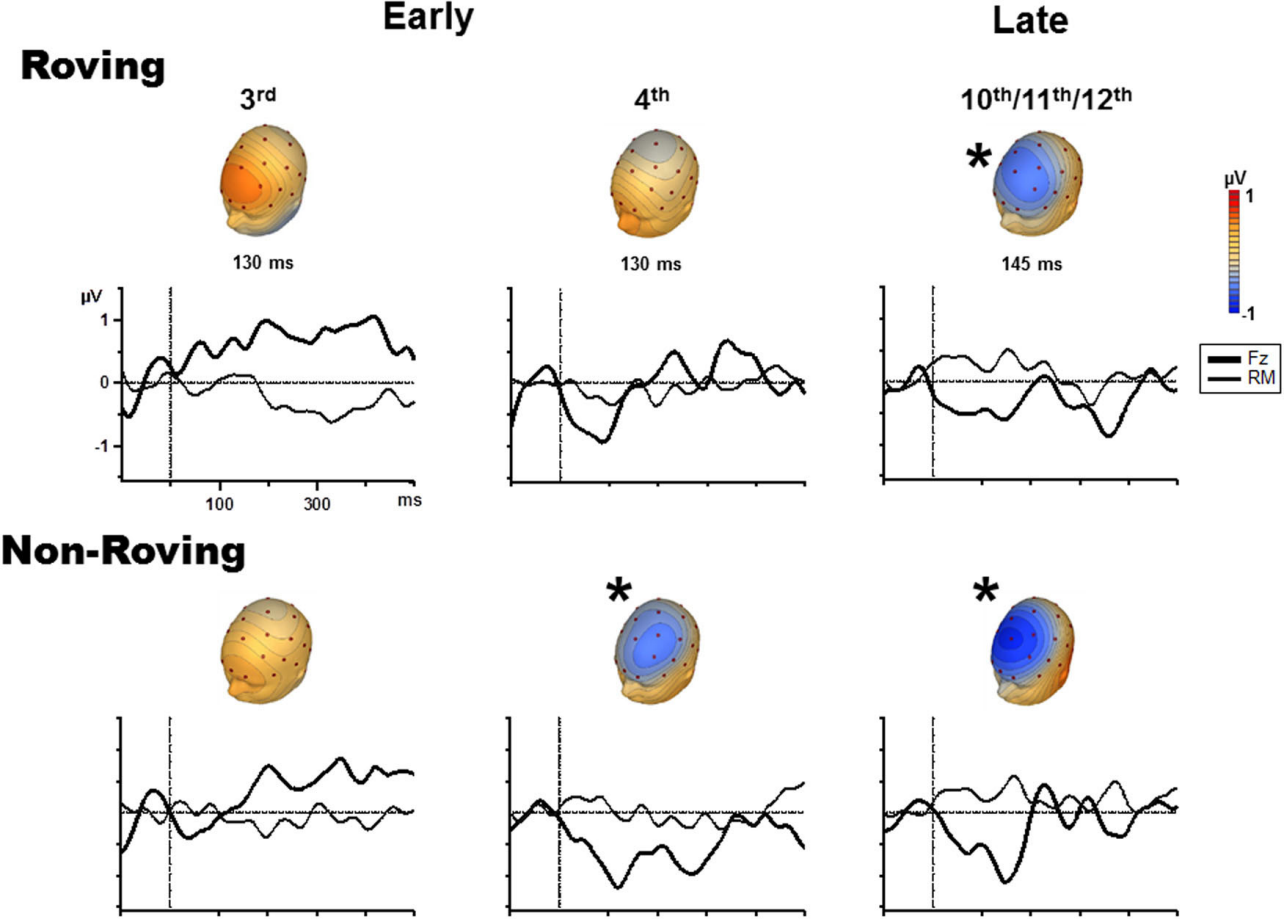
**Difference waveforms (ERP response to the probe minus ERP response to the control) are displayed for the Fz (solid, thick line) and mastoid (solid, thin line) electrode sites for the 3rd position (left column), 4th position (middle column), and late position (right column) in the Roving condition (top row) and Non-Roving condition (bottom row)**. Voltage maps obtained from the difference waveforms at the peak of the measuring interval are presented above each waveform. Significant MMNs are marked with an asterisk, showing typical fronto-central MMN scalp topography.

Figure 3 displays the grand averaged difference waveforms. The voltage maps are shown from the peak of the measuring interval (Table 2) positioned above each graph to display the scalp topography. The scalp topography of the significant MMNs are consistent with typical fronto-central pattern observed for MMN, including the inversion in polarity at the mastoid electrodes (Giard et al., 2014). As there was no a priori expectation for differences in the cortical generators of the MMNs elicited by Roving and Non-Roving conditions, and no apparent differences observed in the voltage maps, source analyses were not performed.

## Discussion

The goal of the current study was to determine whether stimulus context modulated the timing to stream segregation. The key finding was that the regularity of stimulus input led to more rapid stream segregation, as indexed by MMN to intensity probe tones occurring earlier in the tone trains for the Non-Roving (4th position) than the Roving condition (late position). MMNs were elicited by intensity probe tones only in the later part of the tone trains in the Roving condition, suggesting a longer buildup period to neurophysiologic stream segregation for those trains compared to when frequency was repeated from train to train.

These results can be interpreted on the basis of a difference in the timing of the buildup between conditions due to a change in the speed of the segregation process. That is, the elicitation of MMN by the 4th position probe tones occurring only in the Non-Roving condition suggests that the tones were neurophysiologically segregated when the 4th position probe tone occurred. The absence of the MMN to the 3rd position probe tones in the Non-Roving condition suggests that the buildup period was still ongoing at that time. That is, the absence of MMN to the 3rd position probe tone indicates that the silence reset the system but that the speed was faster when the tone frequencies of the trains were repeated. An alternative possibility is that there was no resetting across trains but that the MMN generating system was limited. Although there is evidence that at least two standard tones are required before MMN can be elicited in a single oddball sequence that roves frequencies across tone trains (Cowan et al., 1993), there is no evidence about whether that is also true for oddball sequences that first require segregation to be detected. That is, if the process of detecting the standards occurs only after the segregation occurs, then the MMN system would not have had enough time in our fast paradigm. There may have been a confound between the process of segregation and the standard formation process (Sussman, 2007) in this fast paradigm. We have evidence that the stream segregation process occurs prior to the regularity detection process (Yabe et al., 2001; Sussman, 2005). Thus, if one assumes that the standard formation process begins only once the stream segregation process is neurophysiologically represented, then the standard formation process would begin only after that. These data cannot resolve that issue, and therefore, from these results, we can only conclude that the representation of stream segregation occurred more quickly in the Non-Roving than the Roving condition. Adaptation to the constancy of the frequency presentation could be allowing the segregation process to occur more quickly.

Previous behavioral studies support the explanation of resetting perceived segregation under various conditions (Bregman, 1978; Anstis and Saida, 1985; Beauvois and Meddis, 1997; Carlyon et al., 2001; Cusack et al., 2004; Snyder et al., 2008; Haywood and Roberts, 2010). Although previous behavioral studies have overall suggested that 4 s of silence resets the segregation process (Bregman, 1978; Cusack et al., 2004), Bregman (1978) also demonstrated that shortening the length of the silent period biased the system toward a quicker recovery of segregation. Beauvois and Meddis (1997) showed that varying the silent interval led to an exponential decay of segregation responses as the silence was increased. They presented a 10 s constant frequency induction sequence of a repeating tone at 90 ms SOA and then a test sequence of eight repetitions of two alternating tones with a six semitone frequency separation and the same 90 ms SOA. Between these sequences they varied the silent period from 0 to 8 s. Subjects were instructed to respond after hearing the test sequence and indicate whether they heard an integrated or segregated percept. There was an exponential decrease in the average number of segregation responses as the silent period was increased. This suggested that the time between trains of sounds in their paradigm determined the degree of resetting. However, Beauvois and Meddis (1997) used a constant frequency induction sequence with no silence between the induction and test sequence. Haywood and Roberts (2013), in contrast, showed that an alternating frequency induction sequence had less of an effect on the perception of streaming. Even if the effect was smaller, our neurophysiologic results may be partially explained by their results. The constancy of the Non-Roving condition may have had a similar biasing effect on the buildup as did shortening of the silence between trains. That is, the silence between trains may have shifted the exponential recovery of neurophysiologic segregation. This suggests a possible rectifying explanation, whereby carryover effects allow the auditory system to maintain a neural representation of segregation that shortens the corresponding buildup period by allowing a quicker recovery of segregation in succeeding trains when the environment is Non-Roving for any number of factors that allow for constancy of perceptual organization.

Overall, our results demonstrate that the dynamics of the environment influences the way in which the auditory system extracts regularities from the input. In dynamically changing situations, it would seem that there would be a propensity to maintain the model of the auditory environment that has been ongoing (Bregman, 1990), and therefore, more information would need to be accumulated before the model was altered. Fully resetting the stream segregation process every time a silence occurs may not be advantageous to maintaining a consistent representation of the auditory scene. In contrast, with multiple overlapping properties of the stimulus input, further analysis may be needed to determine whether a putative new piece of information was an adjustment to the current model, or a signaling to the onset of a new “event.” This could alter the timing of segregation for the neurophysiologic model of the auditory scene. To put it into a more realistic context, when walking into a new auditory environment, and successfully organizing the scene (identifying the distinct streams within it), it would be disadvantageous if the scene integrated back every time there was a slight change or silent moment. In contrast, relatively large jumps in frequency from what has been previously ongoing may suggest the onset of a new event, such as a new speaker. Overall, these mechanisms provide the auditory system with an elegant solution to the problem of maintaining stable neural representations in the face of consistency or change, each of which are often encountered in a noisy auditory scene.

### Conflict of interest statement

The authors declare that the research was conducted in the absence of any commercial or financial relationships that could be construed as a potential conflict of interest.
