# Corrigendum: The effect of stimulus context on the buildup to stream segregation

**DOI:** 10.3389/fnins.2014.00280

**Published:** 2014-09-04

**Authors:** Elyse S. Sussman

**Affiliations:** Department of Neuroscience, Albert Einstein College of MedicineBronx, NY, USA

**Keywords:** auditoryperception, mismatchnegativity, streamsegregation, event-relatedpotentials, auditoryscene analysis

One of the funding sources was omitted from the Acknowledgments list. The corrected list is as follows. This research was supported by the National Institutes of Health (R01DC004263), the Army Research Office (58760LS, Elyse S. Sussman) and the MSTP training grant (T32-GM007288, Jonathan Sussman-Fort).

## Conflict of interest statement

The author declares that the research was conducted in the absence of any commercial or financial relationships that could be construed as a potential conflict of interest.

